# Aggressive Behavior, Hostility, and Associated Care Needs in Patients With Psychotic Disorders: A 6-Year Follow-Up Study

**DOI:** 10.3389/fpsyt.2019.00934

**Published:** 2020-01-08

**Authors:** Margo D. M. Faay, Jim van Os, the Genetic Risk and Outcome of Psychosis (GROUP) Investigators, Therese van Amelsvoort

**Affiliations:** ^1^ Department of Psychiatry, University Medical Center Utrecht, Utrecht, Netherlands; ^2^ Department of Psychosis Studies, Institute of Psychiatry, King’s College London, King’s Health Partners, London, United Kingdom

**Keywords:** aggression, violence, needs assessment, psychotic disorders, incidence, risk factors

## Abstract

**Background:** Hostility and aggressive behavior in patients with psychotic disorders are associated with demographic and clinical risk factors, as well as with childhood adversity and neglect. Care needs are an essential concept in clinical practice; care needs in the domain of safety for others reflect the actual problem the patient has. Hostility, aggressive behavior, and associated care needs, however, are often studied in retrospect.

**Method:** In a sample of 1,119 patients with non-affective psychotic disorders, who were interviewed three times over a period of 6 years, we calculated the incidence of hostility, self-reported maltreatment to others and care needs associated with safety for other people (safety-to-others). Regression analysis was used to analyze the association between these outcomes and risk factors. The population attributable fraction (PAF) was used to calculate the proportion of the outcome that could potentially be prevented if previous expressions of adverse behavior were eliminated.

**Results:** The yearly incidence of hostility was 2.8%, for safety-to-others 0.8% and for maltreatment this was 1.8%. Safety-to-others was associated with previous hostility and vice versa, but, assuming causality, only 18% of the safety-to-others needs was attributable to previous hostility while 26% was attributable to impulsivity. Hostility, maltreatment and safety-to-others were all associated with number of unmet needs, suicidal ideation and male sex. Hostility and maltreatment, but not safety-to-others, were associated with childhood adversity. Neither safety-to-others, maltreatment nor hostility were associated with premorbid adjustment problems.

**Conclusion:** The incidence of hostility, self-reported aggressive behaviors, and associated care needs is low and linked to childhood adversity. Known risk factors for prevalence also apply to incidence and for care needs associated with safety for other people. Clinical symptoms can index aggressive behaviors years later, providing clinicians with some opportunity for preventing future incidents.

## Introduction

Most patients with psychosis are not violent but there is an increased risk compared with the general public (1). Aggressive and hostile behavior in patients with psychosis has a major impact on patients’ families, health care workers and society in general, but most of all on patients. Hostile behavior can decrease the quality of life and has been associated with poorer social functioning (2). Several demographic and clinical risk factors have been reported such as male sex, socioeconomic deprivation, cannabis use, suicidality and clinical symptoms such as impulsiveness and excitement (3–8), although there are some differences between these studies.

In addition to demographic and clinical risk factors, factors in childhood may also increase the risk for adult aggressive and hostile behavior. Physical and sexual adversity during childhood was significantly associated with later violence in patients with psychotic disorders (4, 9, 10). Aspects of premorbid social adjustment, such as difficulties in social relationships during the stages of childhood and adolescent development, could also increase the risk of future violent behavior among patients diagnosed with schizophrenia (11). Insight in early risk factors can contribute not only to identifying patients at risk but also to clarifying the pathways leading to violence, opening the way to treatment (12).

While most studies use violence or aggressive incidents as outcome to determine risk factors, these incident rates alone may not reflect the actual problem. The concept of care needs should be considered in addition to incident outcomes. Care needs in the domain of safety for others are essential in clinical practice for both patient and clinician. Care needs allow patients to clarify the impact on their daily lives and the need for care they require to cope with these problems. For the clinician, insight in these needs enables risk management in the domain of safety. Little is known about the relation with other indicators for aggressive behavior and if the established risk factors for violence or aggressive incidents also apply to care needs in the domain of safety for others.

Many studies have identified risk factors and rates for aggressive behavior in patients with psychotic disorders in analyses that were cross-sectional or in retrospect. Prospective data can be more informative for incidence, causality and to indicate the predictive value of risk factors. These data can thus help in the management of hostile and aggressive behaviors.

In this paper, we report the analyses of violence, hostility and associated care needs of the Genetic Risk and Outcome of Psychosis (GROUP) study, a naturalistic multicenter, six-year prospective cohort study in a sample of patients with non-affective psychotic disorders. The aim was to analyze the incidence, persistence, risk factors and attributable fraction for hostility, self-reported aggressive behavior, and associated care needs in a population of patients with psychosis.

## Methods

### Study Design

The GROUP study is described in detail elsewhere (13). A representative cohort of patients aged 16 to 50 years, with a diagnosis of a non-affective psychotic disorder according to the DSM-IV (14), was included. The study also included first-degree family members of these patients and healthy controls, but for the current paper only data from patients were used. In selected representative geographical areas in the Netherlands and Belgium, patients were identified through clinicians whose caseload was screened for inclusion criteria. Subsequently, a group of patients presenting consecutively at these services either as outpatients or as inpatients were recruited. At baseline and then after three and six years, a number of questionnaires, tests and clinical interviews were administered. The study protocol was approved by the Ethical Review Board of the University Medical Centre Utrecht and by local review boards of each participating site. All participants gave written informed consent.

### Measures

#### Outcome Measures

The three main outcome measures were: the hostility item of the Positive and Negative Syndrome Scale [PANSS (15)], maltreatment to others (single question) and the safety-to-others item of the Camberwell Assessment of Need Short Appraisal Schedule [CANSAS(16)].

Hostility is one of the items on the PANSS positive subscale and is defined as “verbal and nonverbal expressions of anger and resentment, including sarcasm, passive aggressive behavior, verbal abuse and assaultiveness”. Thus, hostility can include a relatively mild to moderate expression of violence. All PANSS items are scored on a scale of 1–7. A score of 1 indicates the item is absent, a score of 7 indicates an extreme level of psychopathology. All 30-itmes of the PANSS were administered at the three study visits: baseline, year 3 and year 6.

Safety-to-others is one of the 22 items on the CANSAS and is used to measure care needs associated with safety for other people. The CANSAS allows clinicians and patients together to identify the needs for care and can subsequently assist in care planning. The CANSAS contains the first question of the Camberwell Assessment of Needs [CAN(17)]. The safety-to-others item is assessed with the question: “do you think you could be a danger to other people’s safety?” and is scored, like all CANSAS items, as “no problem”, “need” or “unmet need”. A met need indicates a moderate problem but for which the patient receives some sort of intervention. An unmet need indicates a current serious problem for which no intervention has been initiated. The CANSAS was administered at all three visits.

Maltreatment directed at others was assessed with the question: “did you attack or abuse anybody?”. At baseline, the question was aimed at lifetime maltreatment with a distinction between maltreatment prior and after psychosis onset. At follow-up, patients were asked if they attacked or abused anybody in the past three years (i.e. between the study visits).

#### Other Measures

Next to these three outcome measures, demographic items and known clinical and early risk factors were incorporated in the analysis.

Demographic items were age (in years), sex (0 = male, 1 = female), education (no/primary, lower secondary, lower vocational, higher secondary, higher vocational, university) and ethnicity (0 = white, 1 = other), current employment (yes/no), age of onset psychosis, duration of illness in years, and hospitalization (yes/no) lifetime (baseline) or in the past three years (year 3 and 6). Previous studies found both age and lower education risk factors for violent or aggressive behavior (3, 18).

Clinical risk factors included IQ, cannabis use, other PANSS items, number of CANSAS unmet needs and suicidality. Lower intelligence is linked to violent behavior in the general population and in patients with psychosis (1), although a large meta-analysis found no relationship (4). IQ at baseline and at 3-year follow-up was estimated with use of the 4-subtest version of the Wechsler Adult Intelligence Scale [WAIS-III(19)]. At year 6, IQ was estimated with a 15-min version of the WAIS-III short form (20). Cannabis use is a well-known risk factor for violent behavior (5, 7). Cannabis use in the past 12 months (yes/no) was measured with the Composite International Diagnostic Interview [CIDI (21)] at all three study visits.

As for other PANSS items, we selected items that were previously associated with aggression or incorporated in the same PANSS cluster as hostility: P4 excitement, G4 tension, G8 uncooperativeness and G14 poor impulse control (4, 6, 22, 23). Excitement indicates “hyperactivity as reflected in accelerated motor behavior, heightened responsivity to stimuli, hypervigilance or excessive mood lability”. Tension indicates “overt physical manifestations of fear, anxiety and agitation”. Uncooperativeness is defined as “active refusal to comply with the will of significant others, including the interviewer, hospital staff or family, which may be associated with distrust, defensiveness, stubbornness, negativism, rejection of authority, or belligerence”. Poor impulse control, sometimes called “impulsivity”, is defined as “disordered regulation and control of action on inner urges, resulting in sudden, unmodulated, arbitrary, or misdirected discharge of tension and emotions without concern about consequences”. The PANSS total scores for the positive (P1–P7), negative (N1–N7) and general subscale (G1–G16) are presented to describe the current sample. There are suggestions that unmet needs on the CAN are associated with violence (24). The mean number of care needs was therefore also incorporated as a risk factor. Suicidality is linked to aggressive behavior in earlier work (8). In the current study, suicidality was measured with both the CANSAS, the Community Assessment of Psychic Experiences [CAPE (25)] and with a separate question concerning suicide attempts. The suicidality item on the CANSAS hereafter is called “safety-to-self” (“do you ever have thoughts of harming yourself”) and was scored like other CANSAS items. The CAPE was developed as a self-report scale to measure lifetime psychotic experiences. Suicidality is assessed with the question: “do you ever feel like you do not want to live anymore?” and rated from 0 (never) to 3 (almost always). The CAPE was administered at all three study visits. The clinical interview question exploring suicide attempts was comparable with the maltreatment item: “did you attempt to commit suicide?”. Again, at baseline, the question was aimed at lifetime suicide attempts and at follow-up, patients were asked if they attempted to commit suicide in the past three years (i.e. between the study visits).

Early risk factors incorporated in this analysis included premorbid adjustment problems and childhood adversity. The Cannon-Spoor Premorbid Adjustment Scale [PAS(26)] is aimed at evaluating the level of functioning over several phases of life before the onset of the psychotic illness. A higher PAS score indicates worse premorbid functioning. The PAS was administered at baseline. Childhood adversity events were assessed with the Childhood Trauma Questionnaire 25 item short form [CTQ (27)], which is rated with a Likert scale from 1 (never true) to 5 (very often true). The total score is the mean of the 25 items. Conform previous analyses with this dataset, the scores were dichotomized differentiating a high and low adversity group with the cut-off at the 80^th^ percentile of scores for the healthy control subjects (28). The CTQ was administered once, either at baseline or at 3-year follow-up.

### Data Analysis

Release 7 of the GROUP database was used for the analyses. All analyses were done using Stata 15.1 (29). PANSS hostility scores were dichotomized when used as dependent variable in two categorizations: hostility and high hostility. For hostility, scores of 1 were labelled as “no hostility” and scores >1 as evidence for hostility. However, a PANSS score of 2 indicates “questionable pathology”(15) and is therefore a low threshold to label as “hostile behavior”. The primary outcome measure for this analysis is “high hostility”, in line with earlier work (30). For high hostility, scores of 1 and 2 were labelled as no hostility and scores ≥3 were labelled as high hostility. The continuous PANSS hostility score was used for analysis of hostility as an independent variable. For the CANSAS items safety-to-self and safety-to-others, absence or presence of a need was used, without differentiation between met and unmet needs. Thus, the CANSAS need outcomes were dichotomized as need (met or unmet need) or no need. After preparation of these items, four analyses were performed in order to obtain: 1) incidence rates 2) persistence rates, 3) risk factors and 4) the attributable fraction.

First, we calculated the incidence. Data was set for survival analysis using the stset routine in Stata (29). Failure was defined as a score on CANSAS safety-to-others, maltreatment, hostility or high hostility, occurring for the first time after baseline. Failures already existing at baseline (for example, patients with PANSS hostility = 2 at baseline) were therefore excluded from this analysis. Incidence was calculated using failures in single-failure per subject data, meaning only the first occurrence of one of the outcome measures was used to calculate the incidence. The maltreatment item was defined cumulatively including incidents occurring during lifetime (baseline) or between the study visits (year 3 and 6). Hostility was reported over the past week and CANSAS safety-to-others over the past month. In calculating their incidences, we thus assumed that the hostility and CANSAS safety-to-others scores were representative for the behavior over the past three years.

Second, persistence was measured. Persistence stands for continuation of the item at the next visit. For example, patients with a PANSS hostility score both at year 3 and at year 6. Lagged data were used, indexing the status of the outcome in question at the previous visit. The persistence rates thus indicate the number and proportion of patients that scored on the item at two subsequent visits.

Third, the associations between concurrent risk factors (measured at the same time point) and the four main incidence outcome measures (CANSAS safety-to-others, hostility, high hostility and maltreatment) were calculated. Associations were expressed as hazard ratios (HR) and 95% confidence intervals (CI) from Cox proportional hazard regression analysis. The HR describes the relative increase in the rate of the occurrence of the event in one group compared with the other group (31). Although HR is not the same as Relative Risk, the two measures approach each other when the incidence of the outcome in question is low. All HR were adjusted for age and sex. Other relevant variables were incorporated in the Cox regression model.

Forth, in order to indicate the clinical relevance in terms of preventive potential, the Population Attributable Fraction (PAF) was calculated. The PAF gives an estimation of the proportion of the outcome that could have been prevented if the lagged predictor, i.e. measured at the previous time point, was completely eliminated. For example, the proportion of maltreatment scores that could have been prevented if hostility at the previous visit had been successfully treated. The PAF was calculated from the logistic regression model of incident outcomes with the Stata PUNAF package (32).

## Results

### Sample Characteristics

At baseline, 1,119 patients were included. In year 3, 811 patients participated and at year 6, 662 patients participated. Patient characteristics are presented in **Table 1**. The *DSM-IV-TR* diagnoses of the patients were as follows: schizophrenia and related disorders [*DSM-IV-TR* code 295.x; n = 945 (84%)], other psychotic disorders [*DSM-IV-TR* code 297/298; n = 149 (13%)], and psychotic illness in the context of substance abuse or somatic illness [n = 9 (1%)]. Six patients had a missing diagnosis but fulfilled inclusion criteria, and 11 patients had a final diagnosis of affective psychosis but fulfilled criteria of clinical diagnosis of nonaffective psychosis at study entry; these individuals were retained in the sample assuming subtle diagnostic changes between the time of identification for inclusion and actual assessment that could occur in any patient included in the cohort at any time.

**Table 1:** Demographics.

		Baseline n = 1,119 N(%)	Year 3 n = 811 N(%)	Year 6 n = 662 N(%)
Age				
	Mean (SD)	27.60 (7.98)	30.60 (7.22)	33.62 (7.27)
Sex				
	Male	852 (76.14)	623 (76.82)	504 (76.13)
	Female	267 (23.86)	188 (23.18)	158 (23.87)
Ethnicity				
	White	859 (76.76)	664 (81.87)	551 (83.23)
	Other	260 (23.24)	147 (18.13)	111 (16.77)
Education				
	No/primary	151 (13.49)	59 (7.27)	32 (4.83)
	Lower secondary	341 (30.47)	225 (27.74)	150 (22.66)
	Lower vocational	183 (16.35)	187 (23.06)	182 (27.49)
	Higher secondary	270 (24.13)	196 (24.17)	148 (22.36)
	Higher vocational	98 (8.76)	89 (10.97)	91 (13.75)
	University	43 (3.84)	54 (6.66)	58 (8.76)
	Unknown	33 (2.95)	1 (0.12)	1 (0.15)
Employment				
	Yes	513 (51.25)	395 (55.79)	359 (64.45)
	No	488 (48.75)	313 (44.21)	198 (35.55)
Age of onset psychosis		22.98 (7.92)	22.46 (6.76)	22.39 (6.72)
Duration of illness in years		4.98 (4.46)	8.46 (4.44)	11.57 (4.54)
Hospitalization^a^				
	Yes	792 (78.88)	294 (40.11)	183 (30.76)
	No	212 (21.12)	439 (59.89)	412 (69.24)
PAS score				
	Mean (SD)	1.98 (0.88)	1.96 (.89)	1.94 (.88)
CTQ 80^th^ percentile				
	Yes	335 (44.37)	300 (44.05)	225 (40.98)
	No	420 (55.63)	381 (55.95)	324 (59.02)
IQ				
	Mean (SD)	96.08 (15.33)	99.19 (16.32)	101.69 (17.38)
Cannabis use in past 12 months				
	Yes	417 (38.12)	197 (24.97)	142 (22.15)
	No	677 (61.88)	592 (75.03)	499 (77.85)
CAPE suicidality				
	Never	387 (44.18)	405 (54.51)	339 (56.69)
	Sometimes	366 (41.78)	246 (33.11)	203 (33.95)
	Often	93 (10.62)	68 (9.15)	36 (6.02)
	Almost always	30 (3.42)	24 (3.23)	20 (3.34)
Suicide attempts				
	Yes	228 (22.51)	89 (12.23)	53 (8.88)
	No	785 (77.49)	639 (87.77)	544 (91.12)
CANSAS number of unmet needs				
	Mean (SD)	3.24 (2.90)	2.26 (2.36)	2.41 (2.40)
CANSAS Safety-to-self				
	No problem	844 (84.91)	719 (93.13)	577 (92.77)
	Met or unmet need	150 (15.09)	53 (6.87)	45 (7.23)
CANSAS Safety-to-others				
	No problem	862 (87.16)	740 (96.10)	594 (95.50)
	Met or unmet need	127 (12.84)	30 (3.90)	28 (4.50)
Maltreatment to others				
	Yes	188 (20.04)	64 (9.48)	36 (6.34)
	No	750 (79.96)	611 (90.52)	532 (93.66)
PANSS total				
	Mean (SD)	54.41 (16.80)	46.79 (14.24)	47.22 (15.86)
PANSS positive items P1-P7				
	Mean (SD)	12.70 (5.33)	11.00 (4.52)	11.61 (5.37)
PANSS negative items N1-N7				
	Mean (SD)	13.94 (5.95)	11.75 (5.10)	11.73 (5.18)
PANSS general items G1-G16				
	Mean (SD)	27.90 (8.40)	24.05 (7.18)	23.92 (7.59)
PANSS P7 Hostility (dichotomous)				
	No (1)	856 (81.99)	692 (88.95)	535 (85.19)
	Yes (≥2)	188 (18.01)	86 (11.05)	93 (14.81)
PANSS P7 high Hostility (dichotomous)				
	No (≤2)	945 (90.52)	736 (94.60)	585 (93.15)
	Yes (≥3)	99 (9.48)	42 (5.40)	43 (6.85)
PANSS P7 Hostility (continuous)				
	Mean (SD)	1.31 (.77)	1.17 (.53)	1.26 (.78)
PANSS P4 Excitement				
	Mean (SD)	1.34 (.77)	1.26 (.67)	1.28 (.69)
PANSS G4 Tension				
	Mean (SD)	1.87 (1.04)	1.74 (.97)	1.67 (.95)
PANSS G8 Uncooperativeness				
	Mean (SD)	1.23 (.64)	1.14 (.54)	1.15 (.61)
PANSS G14 Poor impulse control				
	Mean (SD)	1.32 (.76)	1.18 (.58)	1.20 (.60)

PAS, Premorbid Adjustment Scale; CTQ, Childhood Trauma Questionnaire dichotomised with the cut-off at the 80^th^ percentile of scores for the healthy control subjects; CAPE, community assessment of psychic experiences; CANSAS, Camberwell Assessment of Need Short Appraisal Schedule; PANSS, Positive and Negative Syndrome Scale; denominators change due to incomplete data.

### Incidence Rates

**Figure 1:**
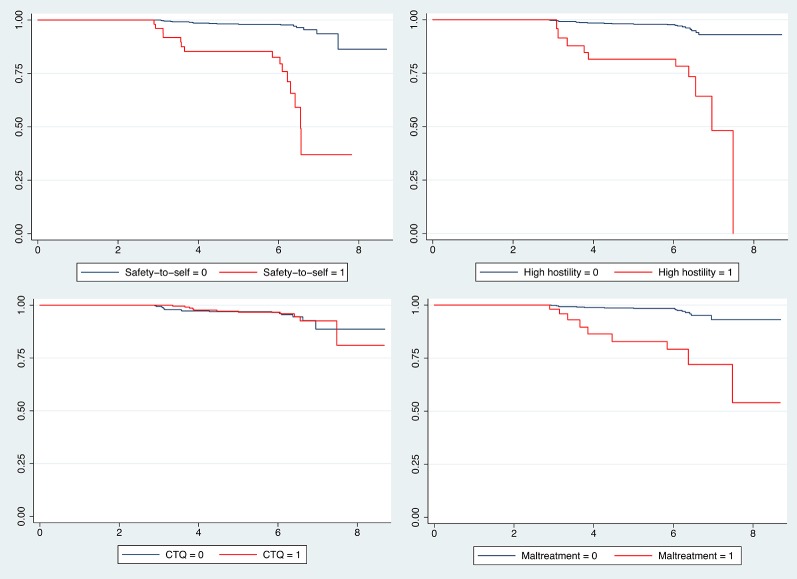
Kaplan–Meier survival estimates for CANSAS safety-to-others. Survival estimates for CANSAS safety-to-others, differentiated by patients with and without high hostility, childhood trauma, maltreatment and CANSAS safety-to-self met or unmet need; X-axis is analysis time in years; CANSAS safety-to-others and safety-to-self item of the Camberwell Assessment of Need Short Appraisal Schedule met or unmet need; hostility item of the Positive and Negative Syndrome Scale with scores >2; CTQ, Childhood Trauma Questionnaire dichotomized with the cut-off at the 80^th^ percentile of scores for the healthy control subjects.

At baseline,127 patients (12.8%) had a CANSAS safety-to-others need. Across the three visits, a total of 162 patients yielded 185 events of CANSAS safety-to-others. From baseline, 32 patients had a new CANSAS safety-to-others in single failure per subject data **Table 2**). The yearly incidence thus was 0.8%. Kaplan-Meier survival estimates for CANSAS safety-to-others, differentiated by patients with and without PANSS hostility, maltreatment, CANSAS safety-to-self and childhood trauma are shown in **Figure 1**.

**Table 2:** Number of failures, person-time and incidence for CANSAS safety-to-others, PANSS hostility, high hostility, and maltreatment to others.

Predictors	Outcome measures																
		CANSAS safety-to-others				PANSS hostility				PANSS high hostility				Maltreatment to others			
		N	Failures	Person-time	Incidence %	N	Failures	Person-time	Incidence %	N	Failures	Person-time	Incidence %	N	Failures	Person-time	Incidence %
Overall		717	32	4,041	.79	679	104	3,748	2.77	739	57	4,142	1.38	665	66	3,679	1.79%
CANSAS safety-to-others																	
	no					649	93	3487	2.67	707	46	3836	1.20	636	52	3425	1.52
	yes					30	9	107	8.44	38	9	132	6.84	28	9	93	9.72
PANSS hostility																	
	no	658	18	3,438	.52									615	46	3164	1.45
	yes	126	13	458	2.84									112	14	390	3.59
PANSS high hostility																	
	no	681	21	3,695	.57									633	51	3,383	1.51
	yes	61	10	201	4.98									53	9	171	5.26
Maltreatment to others																	
	no	646	16	3200	.50	601	81	2944	2.75	660	42	3244	1.29				
	yes	68	9	241	3.74	69	12	243	4.93	78	9	277	3.25				
CTQ 80^th^ percentile																	
	no	352	16	2,018	.79	336	42	1,892	2.22	365	20	2,091	.96	323	26	1,828	1.42
	yes	262	11	1,462	.75	239	49	1,296	3.78	262	26	1,444	1.80	246	31	1,333	2.33
CANSAS safety-to-self																	
	no	674	17	3,611	.47	646	98	3,381	2.90	697	51	3,706	1.38	623	52	3,280	1.59
	yes	74	15	261	5.75	66	6	220	2.72	77	5	269	1.86	68	9	238	3.77

Failures in single failure per subject data; person time is the total time at risk in days for all patients until failure. CANSAS, Camberwell Assessment of Need Short Appraisal Schedule met or unmet need; PANSS, Positive and Negative Syndrome Scale; PANSS hostility scores >1; PANSS high hostility scores >2; CTQ, Childhood Trauma Questionnaire dichotomized with the cut-off at the 80^th^ percentile of scores for the healthy control subjects.

At baseline, 188 patients (18.0%) had a hostility score >1. A total of 300 patients yielded 367 hostility events across the three visits. After baseline, 104 patients had a hostility event in single failure per subject data, yielding a yearly incidence of 2.8%. For high hostility, 99 patients (9.5%) presented this outcome at baseline. A total of 161 patients yielded 184 high hostility events. After baseline, 57 patients had a new high hostility event in single failure per subject data, thus a yearly incidence of 1.4%.

At baseline, 188 (20%) patients had a history of maltreatment of others. Of these 188 patients, 53 (5.6%) had these problems prior to psychosis onset, while 135 patients (14.4%) indicated the maltreatment took place after psychosis onset. A total of 255 patients collectively had 288 maltreatment scores. After baseline, 66 patients had a maltreatment score in single failure per subject data, leading to a yearly incidence of 1.8%. The yearly incidence of maltreatment in patients with a CANSAS safety-to-others was 9.7% versus 1.5% in patients without a CANSAS safety-to-others.

### Persistence Rates

Of the 99 CANSAS safety-to-others events at time point t, 17 (17.2%) had a persistent CANSAS safety-to-others again at the next visit t+1. For hostility, the persistence rate was 27.4% (46 patients) and for high hostility this was 16.1% (13 patients). For maltreatment, 27 patients (17.5%) had a maltreatment score again at the next visit.

### Risk Factors

Maltreatment was significantly associated with CANSAS safety-to-others (HR 6.28 p = 0.00; **Table 3**) and vice versa (HR 4.50 p = 0.00), corrected for CANSAS safety-to-self. CANSAS safety-to-others (HR 3.58 p = 0.00) and maltreatment (1.88 p = 0.04) were significantly associated with hostility. In the Cox regression model for CANSAS safety-to-others, there was an association with the PANSS items poor impulse control (HR 2.38 p = 0.00), hostility (HR 1.56 p = 0.03) and uncooperativeness (HR.53 p = 0.02) but not with excitement (HR 1.11 p = 0.64) or tension (HR 1.11 p = 0.56).

**Table 3:** Risk factors for CANSAS safety-to-others, PANSS hostility, high hostility and maltreatment to others.

		CANSAS safety-to-othersHazard ratio (95% CI)	PANSS hostility Hazard ratio (95% CI)	PANSS high hostilityHazard ratio (95% CI)	Maltreatment to others Hazard ratio (95% CI)
**Demographic items**					
Age		0.95 (0.89–1.01)	0.99 (0.96–1.02)	0.98 (0.94–1.02)	0.89 (0.84–0.93)***
Sex		0.21 (0.05–0.90)*	0.58 (0.34–0.99)*	0.73 (0.36–1.45)	0.51 (0.25–1.04)
Ethnicity		0.58 (0.20–1.68)	0.87 (0.51–1.47)	1.01 (0.52–1.99)	1.42 (0.79–2.55)
Education					
	No/primary	12.04 (1.40–103.28)*	3.99 (1.32–12.00)*	4.64 (.92–23.34)	4.64 (.96–22.33)
	Lower secondary	2.95 (.36–24.31)	1.97 (.75–5.15)	2.58 (.59–11.33)	1.81 (.41–7.90)
	Lower vocational	3.49 (.44–27.58)	1.99 (.77–5.18)	2.48 (.57–10.82)	1.40 (.32–6.20)
	Higher secondary	2.45 (.29–20.64)	2.11 (.80–5.57)	2.23 (.50–9.97)	1.87 (.43–8.19)
	Higher vocational	1.50 (.14–16.71)	1.43 (.50–4.13)	1.12 (.20–6.16)	1.44 (.29–7.18)
	University^a^	1	1	1	1
Maltreatment to others		6.28 (2.69–14.64)***	1.88 (1.02–3.46)*	2.42 (1.17–4.99)*	
CAPE Suicidality		1.86 (1.26–2.75)**	1.36 (1.07–1.74)*	1.46 (1.07–2.01)*	1.82 (1.36–2.43)***
Suicide attempts		4.15 (1.61–10.68)**	1.33 (.68–2.59)	.60 (.18–1.96)	2.27 (1.19–4.33)*
Baseline IQ		1.00 (0.98–1.03)	1.00 (0.99–1.02)	0.99 (0.97–1.01)	0.99 (0.97–1.01)
CTQ 80^th^ percentile		0.98 (0.45–2.13)	1.65 (1.08–2.50)*	1.85 (1.03–3.33)*	1.74 (1.03–2.93)*
PAS		1.11 (0.72 – 1.69)	1.16 (0.92–1.46)	0.95 (0.69–1.30)	1.18 (0.87–1.61)
Cannabis use in the past 12 months		2.04 (0.98–4.26)	1.79 (1.19–2.68)**	2.16 (1.26–3.70)**	2.74 (1.64–4.57)***
CANSAS number of unmet needs		1.30 (1.17–1.45)***	1.17 (1.10–1.26)***	1.12 (1.02–1.23)*	1.28 (1.18–1.40)***
**CANSAS**					
Safety-to-self		15.32 (7.50–31.32)***	0.69 (0.28–1.69)	0.81(0.29–2.22)	1.42 (0.62–3.26)
Safety-to-others			3.58 (1.69–7.62)***	5.43 (2.44–12.09)***	4.50 (1.96–10.34)***
**PANSS**					
Hostility (continuous)		1.56 (1.03–2.34)*			1.07 (0.69–1.65)
Excitement		1.11 (.71–1.74)	1.31 (1.04–1.66)*	1.60 (1.17–2.20)**	1.26 (0.84–1.89)
Tension		1.11 (.79–1.55)	1.16 (.96–1.40)	1.06 (.81–1.38)	1.03 (0.77–1.37)
Uncooperativeness		0.53 (0.31–0.91)*	1.63 (1.32–2.02)***	1.65 (1.24–2.18)***	1.01 (0.63–1.63)
Poor impulse control		2.38 (1.56–3.64)***	1.59 (1.25–2.03)***	1.19 (.85–1.67)	1.44 (0.92–2.23)

*P < 0.05; **P < 0.01;***P < 0.001; ^a^reference category.

Gray areas encompass variables that were included models; all models and items are adjusted for age and sex;

PAS, Premorbid Adjustment Scale; CTQ, Childhood Trauma Questionnaire dichotomized with the cut-off at the 80^th^ percentile of scores for the healthy control subjects; CAPE, community assessment of psychic experiences; CANSAS, Camberwell Assessment of Need Short Appraisal Schedule met or unmet need; PANSS, Positive and Negative Syndrome Scale; PANSS, hostility scores >1; PANSS high hostility scores >2.

CANSAS safety-to-others (HR 1.86; p = 0.00), hostility (HR 1.36; p = 0.01) and maltreatment (HR 1.82 p = 0.00) were associated with CAPE suicidality. However, only CANSAS safety-to-others was associated with CANSAS safety-to-self (HR 15.32; p = 0.00). Hostility (HR 1.65 p = 0.02) and maltreatment (HR 1.74 p = 0.04) were associated with childhood adversity but CANSAS safety-to-others was not (HR 0.98 p = 0.96). Neither hostility, maltreatment nor CANSAS safety-to-others were associated with premorbid adjustment.

### Population Attributable Fraction

The PAF for hostility in predicting CANSAS safety-to-others was 18% (95% CI −0.00 to 0.34), indicating that, assuming causality, 18% of these safety-to-others needs could have been prevented if hostility at the previous visit was eliminated. For high hostility, the PAF was much lower at 6% (95% CI −0.06 to 0.16). The PAF for maltreatment in predicting CANSAS safety-to-others was 23% (95% CI 0.01 to 0.40).

The PAF for CANSAS safety-to-others in predicting both hostility (−0.3%; 95% CI −0.05 to.04) and high hostility (1.3%; 95% CI −0.08 to 0.05) was negligible. For maltreatment in predicting hostility, the PAF was 1.7% (95% CI −0.06 to 0.09) and for high hostility 3.2% (95% CI −0.09 to 0.14).

For maltreatment, 5.6% (95% CI −0.06 to 0.16) was attributable to hostility and −0.3% (95% CI −0.07 to 0.06) to high hostility. 9.3% (95% CI 0.00 to 0.17) of maltreatment was attributable to CANSAS safety-to-others.

### Additional Analysis

In addition to the aim and scope of this paper, some additional analyses were done to examine the strong association of CANSAS safety-to-others with CANSAS safety-to-self and with poor impulse control. For CANSAS safety-to-self, we conducted a survival analysis, a regression analysis and the calculated the PAF.

There were 62 failures of CANSAS safety-to-self in single failure per subject data and the yearly incidence was 1.6%. The yearly incidence rate for CANSAS safety-to-self in patients with a CANSAS safety-to-others was 10% versus 1.4% in patients without a CANSAS safety-to-others. The yearly incidence of CANSAS safety-to-self was not increased for patients with (1.3%) and without (1.7%) hostility. The same applied to patients with (1.5%) and without (1.5%) maltreatment. For patients with poor impulse control, the yearly incidence of CANSAS safety-to-self was 2.6% versus 1.5% in patients without poor impulse control. For CANSAS safety-to-others, the yearly incidence in patients with poor impulse control was 3.4% versus 0.5% in patients without poor impulse control.

CANSAS safety-to-self was significantly associated with CANSAS safety-to-others (HR 6.89 p = 0.00). In the COX regression model of CANSAS safety-to-self with the PANSS items, there were significant associations with poor impulse control (HR 1.73 p = 0.02) and tension (HR 1.32 p = 0.03) but not with hostility (HR 0.69 p = 0.17), excitement (HR 0.83 p = 0.38) and uncooperativeness (HR 1.27 p = 0.26).

The PAF for poor impulse control in predicting CANSAS safety-to-self was 0.6% (95% CI −0.09 to 0.09) while 26% (95% CI 0.05 to 0.43) of CANSAS safety-to-others was attributable to poor impulse control. The PAF for CANSAS safety-to-others in predicting CANSAS safety-to-self was −1.6% (95% CI −0.06 to 0.03) whereas vice versa, 13% (95% CI −0.03 to 0.26) of CANSAS safety-to-others was attributable to CANSAS safety-to-self.

## Discussion

This paper is, to our knowledge, the first survival analysis of hostile behaviors, self-reported incidents of aggression, and associated care needs. Because of the use of these different measures, results provide insight in data on self-reported incidents of aggression (maltreatment), symptoms (PANSS hostility) and associated care needs (CANSAS safety-to-others). We found that the incidence of these behaviors in this cohort is low and largely confirm previous studies considering risk factors such as male sex, lower educational level, suicidal ideation, cannabis use, and more CANSAS unmet needs. Most known risk factors apply also for care needs associated with safety for other people. Childhood adversity was associated with hostility and maltreatment, but not with CANSAS safety-to-others. Premorbid adjustment problems were not associated with aggression towards others. Persistence rates were low for all outcome measures, although around one in five patients present themselves three years later with the same outcome. Moreover, results of the PAF analysis indicate that, assuming causality, both CANSAS safety-to-others and maltreatment can be traced back to hostile and impulsive behavior at an earlier visit, although the preventive potential is not very high.

The incidence for CANSAS safety-to-others, hostility and maltreatment is lower compared with meta-analytic data (4, 33, 34). However, these rates are often difficult to compare due to the differences in populations (for example: outpatients, acutely ill inpatients or forensic populations), the use of different definitions of violence and aggression (varying from verbal aggression to homicide) and differences in data collection (for example: interview-based, incidents reports or legal data). The current study used mostly interview-based data. This should be valid and reliable for measuring violence incidence in adult mental health care (35). There is, however, no objective data to confirm if these data is reliable and there could be an underestimation because of the 3-year time-intervals between the visits.

There are some differences between the outcome measures throughout the analysis. This could be because of the different references periods. While the maltreatment item was aimed at lifetime problems (baseline) or in the past three years (follow-up), the PANSS was aimed at symptoms that occurred in the past week and the CANSAS reference period was the past month. This difference in reference periods is also why patients reported more maltreatment incidents than CANSAS care needs.

The results on risk factors largely confirm previous studies reporting a link between hostility and aggressive behavior with male sex, lower education, cannabis use and more CANSAS unmet needs (3–5, 24). IQ was not associated with the outcome measures, which is consistent with the results of Witt et al. (4).

Premorbid adjustment problems were not associated with the outcome measures. Only one previous study, as far as we are aware, analyzed premorbid adjustment, reporting a significant association between violent behavior and some factors on the PAS, such as peer relationships, but not all (11). Childhood trauma was linked to hostility and maltreatment, but not to CANSAS safety-to-others. Previous studies found an association between childhood adversity and aggressive behavior in patients with psychosis, although the strongest association may be with sexual, and not with physical abuse (4, 9, 10, 18). Another study using the current dataset found hostile behaviors to be on the route from childhood adversity to psychosis (36). Childhood problems are one of the predisposing factors on the possible causal pathways leading to aggression (12). The complex relation between childhood adversity, conduct disorders and violent behavior in schizophrenia may include shared risk factors on a path leading to both violence and schizophrenia (37).

Impulsivity has previously been associated with violence incidents (4, 6) although we only found an association with hostility and CANSAS safety-to-others and not with maltreatment and high hostility. Another relevant finding was that, assuming causality, 26% of the CANSAS safety-to-others failures could be traced back to poor impulse control three years earlier while only 18% of the CANSAS safety-to-others failures could be explained by hostility at the previous visit. Moreover, for uncooperativeness, there was an increased risk for hostility but a decreased risk for CANSAS safety-to-others. Possibly, the association with hostility is simply because higher scores on the PANSS item lack of cooperation include hostile attitudes or behaviors (15). A similar reasoning may apply to excitement, higher scores of which includes agitation, which was significantly associated with hostility but not with CANSAS safety-to-others nor with maltreatment.

Aggressive behaviors towards self and others are interrelated. Previous work found an association between violence risk and suicidal threats, but not with suicidal attempts (4, 8). Witt et al. point out that impulsivity could be the mediator between suicidality and violence and thereby explain why suicidal behaviors are more strongly associated with violence than suicidal ideations (8). The current results also indicate a strong relation between impulsivity, suicidality and aggressive behavior. There are, however, differences between the outcome measures and there is a lack of association of CANSAS safety-to-self with hostility and maltreatment when corrected for CANSAS safety-to-others.

This study has several limitations. The GROUP study was not specifically designed to measure violence incidents. Although we used three different outcome measures as indicators for aggression, the maltreatment item relies on self-report with long time intervals. Patients may have forgotten incidents that occurred between the visits. Moreover, maltreatment was assessed with one question only. We did not have access to incident reports or data pertaining to the criminal justice system. Drop-out during follow-up could be selective. Patients with aggressive behavior could be less likely to participate with interviews or are, for example, incarcerated or admitted to a closed ward. Second, the results of our PAF analysis should be interpreted with some caution. The PAF assumes causality, which is for the current outcome measures not as clear as with, for example, the PAF of smoking in relation to lung cancer. Third, PANSS scores of 2 indicate “questionable pathology; may be at the upper extreme of normal limits”(15) while we label a score of 2 as “hostile”. We therefore used “high hostility” with PANSS scores >2 as the primary outcome measure, next to the, rather sensitive, measure of hostility. The strength of the current study is its prospective, long-term nature and the large sample size, which allowed for a survival analysis.

Several implications for clinical practice can be mentioned. Importantly, the incidence of indicators of aggressive behavior was very low. In addition, a number of known risk factors for mostly prevalent aggressive behavior were confirmed for incidence measures, which could help in detecting patients at risk. The strong association between CANSAS safety-to-others and safety-to-self stands out, as do associations with treatable psychopathology. At least part of the CANSAS safety-to-others was attributable to previous hostile and impulsive behavior. Under the assumption of causality, if these behaviors been successfully treated three years earlier, the CANSAS safety-to-others may have never occurred. This indicates the importance for clinicians to anticipate on these behaviors in order to prevent future problems in the domain of safety for others, although the preventive potential is not very high.

While the current study contributes to knowledge about aggressive behavior, it remains unclear why some of the outcome measures have significant associations with known risk factors while others have not. Future research should focus on the mechanisms leading to violence and aggression, including the relation with impulsivity and suicidal behavior. Ideally, there should be a combination of self-report, family and clinical observations and legal data.

In conclusion, this paper is, to our knowledge, the first survival analysis of hostile behaviors, self-reported incidents of aggression and associated care needs. Many of the known risk factors for prevalence also apply to incidence and for care needs associated with safety for other people. There were, however, differences between the outcome measures. Violence and aggression in patients with psychosis appears to be a complex concept with different origins, as this and other studies have shown. We found childhood adversity but not premorbid problems to be associated with aggressive behavior. Clinical symptoms can index aggressive behavior and associated care needs years later, providing opportunities for preventive strategies and a possible decrease of incidents of aggression.

## Data Availability Statement

The datasets for this article are not publicly available because: There are legal and ethical restrictions for sharing the dataset during this study. During ethical approval, it was thought that the data might contain potentially identifying or sensitive patient information. Therefore, the authors do not have consent from participants to share the datasets. The ethics committee imposing these restrictions is the Utrecht UMC Ethics Committee, bound by the European Union AVG law. In this situation, the authors are legally bound to restrict the availability of data, although Utrecht UMC can make them available upon request and subject to a data sharing agreement. Requests for this data of the GROUP study can be sent to the first author.

## Ethics Statement

The GROUP study was reviewed and approved by Ethical review board University Medical Center Utrecht. The patients/participants provided their written informed consent to participate in this study.

## Author Contributions

GROUP investigators coordinated the recruitment and data collection. MF and JvO designed the study, carried out the analysis and wrote sections of the manuscript. All authors reviewed and revised the manuscript and approved the final version.

## Funding

The infrastructure for the GROUP study is funded through the Geestkracht programme of the Dutch Health Research Council (Zon-Mw, grant number 10-000-1001), and matching funds from participating pharmaceutical companies (Lundbeck, AstraZeneca, Eli Lilly, Janssen Cilag) and universities and mental health care organizations (Amsterdam: Academic Psychiatric Centre of the Academic Medical Center and the mental health institutions: GGZ Ingeest, Arkin, Dijk en Duin, GGZ Rivierduinen, Erasmus Medical Centre, GGZ Noord Holland Noord. Groningen: University Medical Center Groningen and the mental health institutions: Lentis, GGZ Friesland, GGZ Drenthe, Dimence, Mediant, GGNet Warnsveld, Yulius Dordrecht and Parnassia psycho-medical center The Hague. Maastricht: Maastricht University Medical Centre and the mental health institutions: GGzE, GGZ Breburg, GGZ Oost-Brabant, Vincent van Gogh voor Geestelijke Gezondheid, Mondriaan, Virenze riagg, Zuyderland GGZ, MET ggz, Universitair Centrum Sint-Jozef Kortenberg, CAPRI University of Antwerp, PC Ziekeren Sint-Truiden, PZ Sancta Maria Sint-Truiden, GGZ Overpelt, OPZ Rekem. Utrecht: University Medical Center Utrecht and the mental health institutions Altrecht, GGZ Centraal and Delta).

## Conflict of Interest

The authors declare that the research was conducted in the absence of any commercial or financial relationships that could be construed as a potential conflict of interest.

## Genetic Risk and Outcome of Psychosis (GROUP) Investigators

The GROUP investigators are: Therese van Amelsvoort^g^, Agna A. Bartels-Velthuis^a^, Richard Bruggeman^a,c^, Wiepke Cahn^d,k^, Lieuwe de Haan^f,m^, Frederike Schirmbeck^f,m^, Claudia J.P. Simons^g,i^, Jim van Os^d,j^

^a^ University Medical Center Groningen, University Center for Psychiatry, Rob Giel Research Center, University of Groningen, Groningen, Netherlands;

^c^ Department of Clinical and Developmental Neuropsychology, University of Groningen, Groningen, Netherlands;

^d^ University Medical Center Utrecht, Department of Psychiatry, Brain Centre Rudolf Magnus, Utrecht University, Utrecht, Netherlands;

^f^ Department of Psychiatry, Amsterdam UMC, University of Amsterdam, Amsterdam, Netherlands;

^g^ Department of Psychiatry and Neuropsychology, School for Mental Health and Neuroscience, Maastricht University Medical Center, Maastricht, Netherlands;

^i^GGzE Institute for Mental Health Care, Eindhoven, Netherlands;

^j^ Department of Psychosis Studies, Institute of Psychiatry, King’s College London, King’s Health Partners, London, United Kingdom;

^k^ Altrecht, General Mental Health Care, Utrecht, Netherlands;

^m^ Arkin, Institute for Mental Health, Amsterdam, Netherlands
